# Leadership, power, and subjectivation in global nursing: the trajectory of Doris Grinspun

**DOI:** 10.1590/1980-220X-REEUSP-2026-0192en

**Published:** 2026-11-06

**Authors:** Amalia Graciela Silva Galleguillos, Oscar Javier Vergara Escobar, Ingredy Nayara Chiacchio Silva, Deybson Borba de Almeida, Gilberto Tadeu Reis da Silva

**Affiliations:** 1 Universidad de Chile, Santiago, Chile.; 2 Universidad El Bosque, Bogotá, Colombia.; 3 Universidade Federal da Bahia, Salvador, BA, Brazil.; 4 Universidade Estadual de Feira de Santana, Feira de Santana, BA, Brazil.

**Keywords:** Nursing, Leadership, Biography, History of Nursing

## Abstract

This is a historical study with a biographical focus that used the Foucauldian framework as the underlying theory for data analysis.

**Objective:**

To analyze the professional and political trajectory of Doris Grinspun, highlighting her contributions to Canadian and international nursing in light of the Foucauldian concepts of power, knowledge, and subjectivation.

**Method:**

Historical study with a qualitative approach and a biographical focus. Data were produced through a semi-structured interview, conducted in a virtual setting, and submitted to thematic content analysis. The interpretation was grounded in the underlying theory of the concepts of technologies of the self, power-knowledge, governmentality, and regimes of truth, as proposed by Michel Foucault. The study met the criteria of the Consolidated Criteria for Reporting Qualitative Research and current ethical precepts.

**Results:**

The trajectory reveals processes of subjectivation constituted since childhood, mediated by family, academic, and professional experiences that fostered the construction of ethical and political leadership. Sociopolitical engagement, the articulation between practice, education, and evidence, and strategic action in professional organizations with international repercussions stand out. Conclusion: Her leadership configures an ethical-political phenomenon that strengthens the governmentality and global visibility of nursing.

## INTRODUCTION

Nursing, as a social and professional practice, has been historically constituted as a space of symbolic and material disputes crossed by relations of power, knowledge, and identity. From Michel Foucault’s perspective, subjects are not isolated autonomous entities, but emerge amid apparatuses of power and technologies of the self that shape ways of being and acting inscribed in the social fabric^(1,2)^. Understanding the trajectories of nursing leaderships, therefore, goes beyond individual biographical analysis: it requires recognizing historical, discursive, and institutional processes that shape subjectivities and reconfigure professional rationalities.

Research on militant nurses shows that political-professional engagement arises from the tension between alienation and emancipation and between subalternity and protagonism^(3)^. Nursing, marked by historical invisibility and ties to biomedical and religious models, finds in institutional leadership and militancy pathways to affirm its collective identity and expand its social relevance^(4)^. In these trajectories, discursive practices, educational processes, and institutional rationalities are articulated, configuring specific modes of subjectivation and favoring the emergence of leaderships capable of contesting the meanings of the profession and influencing structuring transformations^(5)^.

In this scenario, the trajectory of Doris Grinspun in the Canadian context proves singular. Her work transcends clinical practice and configures an ethical-political project to strengthen nursing as a critical, autonomous profession committed to social justice. By leading debates on public policy, professional education, care quality, and institutional representation, Grinspun exemplifies the Foucauldian political subject: one who acts upon others, establishes power relations, and, simultaneously, constitutes herself through practices of freedom. Her biography makes explicit the articulation between technologies of the self and governmentality strategies that make it possible to reorient nursing institutions, discourses, and practices on a national and international scale.

Doris Grinspun was born in Chile in 1955, into a family of Jewish roots who later emigrated to Israel, where she began her academic education. In her personal life, Doris is married to Dr. Elliot Grinspun, and the couple has children, maintaining a solid family foundation that accompanied her professional transition and consolidation in Canada from the 1980s onward.

Doris Grinspun’s life story links her figure to that of a global health strategist. Her greatest contribution is the movement for nursing to make decisions based on the best available scientific evidence, raising the status of the profession and the quality of patient care.

Examining her life story makes it possible to situate Canadian nursing within a broader horizon of political and professional struggles, articulating education, associative engagement, and confrontation with hegemonic models of care^(6,7)^. This means understanding that Grinspun’s trajectory is not an individual narrative, but the expression of social and professional processes that seek to overcome alienation, consolidate collective identities, and affirm nursing as an ethically oriented social practice. This Foucauldian perspective sheds light on how regimes of truth, political-professional rationalities, and subjectivities that sustain transformations in the health field are produced^(8)^.

Thus, the objective of this article is to analyze the trajectory of Doris Grinspun, highlighting her contributions to Canadian and international nursing and situating her militancy and leadership as central elements in the profession’s processes of transformation. The analysis is guided by the following question: In what way did Doris Grinspun’s life trajectory and political militancy contribute to the constitution of Canadian nursing as a social, ethical, and civic practice, and what apparatuses of power and knowledge, in light of the Foucauldian framework, are evidenced in her professional and institutional work? It is hoped that this will contribute to the scientific debate on identity, politics, and power, offering theoretical-analytical elements to understand the role of contemporary leaderships in consolidating nursing as a field of knowledge, practice, and social intervention.

## METHOD

### Type of Study

This is a historical study with a qualitative approach and a biographical focus on the professional trajectory of the nurse Doris Grinspun. Within qualitative research, this type of approach makes it possible to explore, discover, and assess how people, through their experiences, understand their past, link it to the social context, and give it meaning in the present^(9)^. These methodological possibilities converge with one another: the Foucauldian theoretical-philosophical framework, historical research, and the biographical focus are theoretical perspectives that add greater depth to qualitative research, the purpose of which is critical, robust analysis of the data rather than the ability to generalize the findings.

In this direction, to understand the biography in question, the Foucauldian philosophical framework was used, which addresses the constitution of subjects and points to the theoretical aspects of technologies of the self, divided into four main groups: (1) techniques of production, which permit the production, transformation, or manipulation of things; (2) techniques of sign systems, which permit the use of signs, meanings, symbols, or signification; (3) techniques of power, which determine the conduct of individuals and subject them to certain ends or domination, objectifying the subject; (4) techniques of the self, which permit individuals to perform, by their own means or with the help of others, a certain number of operations on their own bodies, souls, thoughts, conduct, and way of being, so as to transform themselves in order to attain a state of happiness, purity, wisdom, perfection, or immortality^(2)^.

### Population

In order to carry out a historical reconstruction of the subject of the biography, a semi-structured interview was used as the data collection technique, conducted in Spanish, the mother tongue of the interviewee for the production of the oral source.

### Data Collection

The interview took place virtually, through the online platform Google Meet. Data collection was carried out between August and September 2021, lasting approximately 1 hour and 20 minutes, ensuring adequate conditions for recording and preserving the narrative.

### Data Analysis and Processing

For data analysis, the thematic content analysis method was chosen, which included the following steps: selection of registration units, enumeration based on frequency, treatment of results, and interpretation. It is worth noting that this choice adds the possibility of a more in-depth data analysis mediated by an underlying theory concerned with the analysis of the subject. The categorization of the data based on a theoretical-philosophical framework was grounded in the registration units and manifest content. The analysis was not limited to the frequency of the recording units, but prioritized the interpretation of discursive practices, power relations, and processes of subjectivation.

The analytical positioning was based on Foucault’s work, with pre-established categories, while allowing room for the idiosyncrasies that would emerge over the course of the data analysis. The emerging categories were subsequently compared against the Foucauldian framework, in an analytical structure that made it possible to identify apparatuses of power, technologies of the self, and modes of subject constitution. In the data organization stage, the *software* Qualitative Data Analysis WebQDA® was used for support, making it possible to systematize and explore the interview content in depth^(10)^.

### Ethical Aspects

This research is part of the parent project entitled “Nursing Management Models: nurses’ memories,” approved by the Research Ethics Committee under Opinion No. 5.462.599, with all regulations governing research with human subjects being respected. The interview only took place upon the interviewee’s authorization and signature of the copyright assignment form, and was subsequently transcribed and validated by the participant herself before being used for analysis^(11)^. Statements from the interview are identified by the letters “ENT,” followed by the interviewee’s initials “DG.”

The entire process was guided by the *Consolidated Criteria for Reporting Qualitative Research* (COREQ), Portuguese-language version, which establishes criteria for reporting qualitative research, ensuring transparency, rigor, and reproducibility^(12)^.

Artificial intelligence was used in a focused manner to assist in writing and structuring the text.

## RESULTS

The narratives reveal a trajectory permeated by early practices of collective organization, which outline modes of subjectivation linked to the exercise of leadership. Since childhood, the subject of the biography describes the formation of structured groups, endowed with norms and devices of belonging, which signals an early orientation toward peer coordination and the management of shared matters.

The discourses presented in Chart 1 situate these experiences along three axes: the analysis of discourse, practices, and projects in nursing education. Based on these accounts, Chart 2 gathers the analytical categories and details the Foucaultian processes mobilized to interpret the subject’s trajectory.

**Chart 1:** Discourse, practices, and projects of Doris Grinspun – Salvador, BA, Brazil, 2026.

Discourse analysis in nurse education	
Discourse of leadership experiences	*Yo formé el club de las pulguitas – risas – (…) teníamos carnet que los plastificamos con las velitas. (ENT-DG)*
	*Ellas formaron su grupo… y yo dije ‘¿Y el resto de nosotras qué vamos a hacer?’. Club de la pulguita, y obviamente si lo vamos a hacer, lo vamos a hacer mejor. (ENT-DG)*
	*Después me hice líder en el movimiento social (…) fui a Uruguay a hacer un curso de líder de juventud y me transformé en una de las líderes del movimiento Barclays. (ENT-DG)*
	*Siempre en cada país que estuve empecé como enfermera clínica porque quería entender la práctica del país (…) y lo segundo que hice fue obtener un título académico en cada país. (ENT-DG)*
Discourse of family experiences	*Desde los 6 años tenía que decir algo sobre lo que el diario decía en la comida… y los viernes teníamos que cantar. (ENT-DG)*
	*Nos educaron a ser libres… mi padre quería que sus hijas vuelen. (ENT-DG)*
	*Imagínate… estaba al otro lado del mundo, en un país de guerra, con un idioma que no conocía bien, y mis padres solo decían ‘que te vaya bien’. (ENT-DG)*
Discourse of the academic process	*Una cosa es saber el idioma y otra cosa es hablarlo… cuando llegué todo era una línea y yo no entendía nada. (ENT-DG)*
	*Te hicieron buena enfermera, muy disciplinada, pero había cosas absurdas… te hacían separar tus problemas de los pacientes. (ENT-DG)*
	*Me dijeron que abra una unidad para soldados con lesiones… yo dije ‘soy estudiante’, pero respondieron ‘dijeron que tú podías hacerlo’. Me miré al espejo y dije ‘si ellos dicen que puedo, será porque puedo’. (ENT-DG)*
**Practice analysis in nurse education**	
Nursing work practices	*Si al centro de tu atención están los pacientes, nunca vas a hacer algo mal. (ENT-DG)*
	*Revolucioné todo el hospital sin saber que lo estaba haciendo, porque pensaba primero en los pacientes. (ENT-DG)*
	*Yo vine a la profesión porque me enamoré de la gente… la enfermería es una herramienta para ayudar al prójimo. (ENT-DG)*
	*Lo que dije fue: gracias, gracias por haber trabajado… y por favor quédense, las crisis no duran para siempre. (ENT-DG)*
Nursing practice during the pandemic	*La pandemia nos dio en bandeja la oportunidad de lucirnos como profesión… pero no hubo un poder coordinador internacional. (ENT-DG)*
	*Si se nos hubiera escuchado, estaríamos mil veces mejor. (ENT-DG)*
Sociopolitical engagement practices	*Trabajar en la guerra me hizo pacifista y activista de la paz… eso no se olvida nunca. (ENT-DG)*
	*Buscaba la justicia humana… siempre fue mi intención cambiar el mundo. (ENT-DG)*
	*El poder no sirve para nada si no se comparte… mientras más lo compartes, mejor para todos. (ENT-DG)*
**Project analysis in nurse education**	
Nursing: perspectives on present and future	*Si los estudiantes salen con la expectativa de practicar basado en la evidencia, ganamos. (ENT-DG)*
	*La mejor oportunidad es investigación centrada en la práctica, no artículos por artículos. (ENT-DG)*
Landscape of best practices in nursing	*Es un movimiento social de conocimiento. (ENT-DG)*
	*Cuando llegué teníamos 9.000 miembros… ahora somos 46.000. (ENT-DG)*
	*Nosotros damos metodología y materiales; ellos nos dan cultura, liderazgo y riqueza humana… aprendemos juntos. (ENT-DG)*
Impact of the program on people’s lives	*Cuando veo emails de gente que ni conozco que me dicen la diferencia que hice en sus vidas… eso es lo más importante. (ENT-DG)*

Source: Prepared by the authors, 2026.

**Chart 2:** Synthesis of analytical categories – Salvador, BA, Brazil, 2026.

Analytical category	Core content	Relevant excerpt(s)	Foucauldian processes mobilized	Interpretive synthesis
Leadership experiences	Early formation, peer organization, youth leadership courses	*“Yo formé el club de las pulguitas…”*	Governmentality apparatuses; technologies of the self	Leadership emerges from early practices of organization and recognition
Family experiences	Autonomy, critical debate, emancipatory education	*“nos empujaban la máquina del pensar y del cuestionar”*	Technologies of the self	Family as a space that shapes critical and ethical subjectivity
Academic process and discipline	Linguistic barriers, rigorous training, cultural displacement	*“te hicieron buena enfermera… disciplinados pero absurdos”*	Discipline; regimes of truth	Training shapes the professional body through discipline and resistance
Clinical practice and the ethics of care	Patient-centered care, institutional impact	*“si al centro de tu atención están los pacientes…”*	Power-knowledge; governance of care	Care underpins ethical and transformative leadership
Pandemic and political disputes	Lack of being heard, global crisis, untapped potential	*“si se nos hubiera escuchado…”*	Discursive disputes; biopolitics	The pandemic reveals tensions between visibility and political limitation
Sociopolitical engagement	Peace activism, human justice	*“me hizo pacifista y activista de la paz”*	Practices of freedom; resistance	Political engagement guides ethical and collective leadership
Projects for the future	Evidence-based education, practice-research integration	*“investigación centrada en la práctica”*	Production of knowledge; governmentality	The future depends on the articulation between practice, research, and ethics
Best practices and expansion	International program, co-learning, impact on patients	*“es un movimiento social de conocimiento”*	Power-knowledge networks; internationalization	The program as a global apparatus for strengthening nursing

Source: Prepared by the authors, 2026.

## DISCUSSION

Doris Grinspun’s professional and academic trajectory reveals a singular path in which life experiences, educational investment, clinical practices, and institutional work mutually constitute one another. To understand it, it is necessary to analyze it as the product of historical, discursive, and political relations that run through contemporary nursing. From this perspective, we adopt the Foucauldian framework as our interpretive axis, which makes it possible to understand the formation of Grinspun’s leadership as a process of subjectivation and her political and institutional work as an expression of techniques of power and governmentality. Here we adopt the Foucauldian reading as an analytical framework that integrates empirical evidence – discursive segments from the interview – and conceptual categories (disciplinary technologies, technologies of the self, regimes of truth, power-knowledge, governmentality), in order to make explicit how practices and discourses shape subjects and professional practices. The discussion is organized into four analytical blocks: (1) the impact of family context and professional socialization; (2) the transformation of nursing education in Latin America; (3) innovations in research, curriculum, and simulation; and (4) institutional leadership and advocacy – governmentality, resistance, and expansion of the profession.

### Impact of Family Context, Socialization, and the Production of Nursing Subjects

Grinspun’s early trajectory indicates that family values, transnational mobility, experiences of confrontation, and encouragement toward education constituted elementary apparatuses of subjectivation. According to Foucault^(1)^, the subject is not an autonomous origin, but the effect of practices, discourses, and power relations that shape ways of being, acting, and thinking^(2)^. Thus, the experiences reported by Grinspun – early leadership, social awareness, exposure to conflict contexts, and critical education – function as discursive matrices that produced a professional oriented toward ethics, collective responsibility, and political engagement. More precisely, these family and formative practices operate as “technologies of the self”: procedures by which the individual constitutes herself, through exercises of judgment, discipline, and care of the self, internalizing norms and developing capacities for political and ethical action.

The literature on professional socialization confirms that childhood experiences, geographic displacement, and situations of vulnerability influence the constitution of professional identities committed to social justice^(13,14)^. Authors point out that biographical trajectories frequently articulate family educational practices and moral formation – a dimension explicitly present in Grinspun’s account^(15)^. From a Foucauldian standpoint, childhood and youth here are not mere temporal antecedents, but historical apparatuses that inscribe modes of subjectivation: they produce predispositions toward critique, toward the responsive exercise of autonomy, and toward collective action. Thus, Grinspun’s constitution as a political subject is anticipated by a network of practices that articulate freedom, discipline, and ethical commitment, which will later be mobilized within the institutional and discursive scope of nursing.

### Transformation of Nursing Education in Brazil and Latin America

The Foucauldian analysis sheds light on macrostructural processes that run through professional education. In Latin America, the field of education is strained by inequalities, curricular fragmentation, and heterogeneity of standards^(16)^. Grinspun’s work is inserted into this context as a force that seeks to shift hegemonic teaching rationalities, bringing education and clinical practice closer together on the basis of international evidence^(17)^. These shifts configure operations of educational governmentality: they set out norms, metrics, and procedures that guide the conduct of students, faculty, and administrators, while at the same time producing governable subjects (professionals trained according to criteria of efficiency and safety).

The advocacy for qualified, integrated, competency-centered education engages with the national debate on the link between teaching and service and the need for critical, reflective curricula^(18)^. On the Foucauldian horizon, the diffusion of these rationalities implies the constitution of educational regimes of truth – sets of statements and practices that define what is considered ‘*valid knowledge*’ and ‘*good practice*’ in education. Thus, Grinspun’s influence transcends mere technical improvement of content: she reconfigures the discursive regime that determines what should be taught, how it should be assessed, and what kind of professional is meant to be trained.

### Innovations in Research, Curriculum, and Simulation: Practices of Knowledge and the Production of Truth

The innovations promoted by Grinspun – curriculum development, applied research, and the use of realistic simulation – articulate practices of knowledge and regimes of truth that structure contemporary nursing. For Foucault, scientific discourses are central mechanisms in the production of truth, capable of legitimizing practices and guiding conduct. In this sense, simulation and practice-centered research do not merely transmit technical competencies; they constitute spaces where statements of truth about care are tested, validated, and disseminated, producing subjects who incorporate this knowledge into their everyday practices.

The creation and implementation of the Best Practice Guidelines (BPGs) represent a paradigmatic example of a power-knowledge apparatus: the BPGs articulate science, policy, and practice by translating evidence into standards and recommendations that standardize conduct, reduce variability in care, and guide clinical decisions. From a Foucauldian perspective, these are technologies that discipline practices while also potentially promoting professional autonomy – because they provide a technical framework that enables evidence-informed clinical judgment. In this way, the BPGs play a dual role: they discipline through regulations and control instruments, and they emancipate by offering resources for decision-making based on technical and ethical criteria. This ambivalent character deserves analytical attention: neither merely oppressive nor purely liberating, these guiding practices inscribe possibilities for agency within institutional limits.

### Institutional Leadership and Advocacy: Governmentality, Resistance, and Expansion of the Profession

At the institutional level, Grinspun demonstrates leadership aimed at transforming structures and practices. Her work at the *Registered Nurses’ Association of Ontario* (RNAO) exemplifies the ability to articulate policies, mobilize groups, and influence public agendas – practices that can be framed as forms of professional governmentality. According to Foucault, governmentality refers to the techniques and rationalities through which the conduct of populations and institutions is directed; in this sense, the development of programs, networks, and advocacy strategies through the RNAO constitutes a process of directing professional conduct and health policy.

The literature indicates that professionalization requires leaderships capable of contesting the meanings of practice and promoting social justice^(19,20)^. Grinspun works in this field by strengthening networks and expanding the visibility of nursing in political arenas, as evidenced during the pandemic and in campaigns for justice in vaccine distribution. Her action also illustrates the Foucauldian concept of resistance: not as mere denial of power, but as the production of alternatives – of distinct forms of organization, knowledge, and political intervention that reconfigure the space of institutional possibilities.

Grinspun’s statement that experiences of war turned her into “*a peace activist*” can be read as a practice of the ethical constitution of the subject: an exercise in technology of the self that guides public choices and modes of collective engagement. Thus, her institutional trajectory operates simultaneously as the production of knowledge, the exercise of power, and the production of subjectivity – a nexus that exemplifies how leadership, political intervention, and the production of knowledge intertwine to expand the governmentality of nursing.

From this perspective, the Foucauldian analysis of Doris Grinspun’s trajectory reveals that her power as a leader stems from the articulation between processes of subjectivation (technologies of the self), disciplinary apparatuses (protocols, curricula, BPGs), and governmentality strategies (institutional actions and advocacy). This articulation shows that power, in contemporary nursing, is exercised not merely as coercion, but as production: of knowledge, of modes of conduct, and of political subjects capable of intervening in the structures that produced them.

Therefore, Grinspun’s biography constitutes heuristic material for understanding how professional agents can institute discursive and institutional shifts that expand margins of action, redefine regimes of truth, and promote changes in health education and policy. From a practical standpoint, this reading suggests that education and management policies should recognize the dual dynamic of these apparatuses (disciplinary/emancipatory) and invest in strategies that foster both the production of applicable evidence and the development of technologies of the self that sustain ethical and critical practice.

The limitations of this study stem from its historical-biographical design centered on the narrative of a single leader, produced through a virtual interview and interpreted exclusively in light of the Foucauldian framework, which circumscribes the analytical scope, restricts the triangulation of sources, and limits the generalization of the findings to other sociopolitical contexts of global nursing.

## CONCLUSION

The Foucauldian analysis of Doris Grinspun’s trajectory reveals that her leadership transcends individual achievements and is configured as an apparatus of transformation capable of reorganizing ways of being, acting, and thinking about nursing from a global perspective. By articulating clinical practice, systems management, applied research, educational innovation, and political activism, Grinspun operates at the intersection of power and knowledge, contributing to reconfiguring health education, practices, and policies.

Her legacy shows that nursing expands its governmentality when it occupies strategic positions in policy formulation, evidence production, and the defense of agendas oriented toward the common good. This work sheds light on pathways that are especially relevant for countries of the Global South, showing that the expansion of professional autonomy depends on leaderships capable of critically intervening in the hegemonic rationalities that shape health systems.

By highlighting how disciplinary apparatuses, technologies of the self, and governmentality strategies are articulated in the constitution of her leadership, this study reaffirms biography as a powerful methodological tool for understanding processes of professional change. Doris Grinspun’s trajectory, marked by ethical commitment, strategic vision, and political engagement, stands as an international reference for building critical, innovative, and transformative leaderships. Finally, it is shown that leadership in nursing is an ethical-political phenomenon capable of producing new regimes of truth, transgressing institutional boundaries, and projecting expanded horizons for the profession on a global scale.

Finally, the study presents as a limitation the absence of triangulation of the results with other sources. However, there is a need to explore, with greater analytical depth, the life history of the subject of the biography based on the interviews. This approach represents an opportunity to apply Michel Foucault’s philosophical framework, exploring technologies of the self in the construction of the subject.

## DATA AVAILABILITY

The full dataset supporting the results of this study has been published in the article itself.
