# Supplementary material for: Leadership, power, and subjectivation in global nursing: the trajectory of Doris Grinspun
Source: Rev Esc Enferm USP. 2026 Nov 6;60:e20260192. doi: 10.1590/1980-220X-REEUSP-2026-0192en (PMC13743177; doi:10.1590/1980-220X-REEUSP-2026-0192en)
Supplement: Translation

# Translation: Liderança, poder e subjetivação na enfermagem global: trajetória de Doris Grinspun

**DOI:** 10.1590/1980-220X-REEUSP-2026-0192pt

**Authors:** Amalia Graciela Silva Galleguillos, Oscar Javier Vergara Escobar, Ingredy Nayara Chiacchio Silva, Deybson Borba de Almeida, Gilberto Tadeu Reis da Silva

**Affiliations:** 1 Universidad de Chile, Santiago, Chile.; 2 Universidad El Bosque, Bogotá, Colombia.; 3 Universidade Federal da Bahia, Salvador, BA, Brasil.; 4 Universidade Estadual de Feira de Santana, Feira de Santana, BA, Brasil.

**Keywords:** Enfermagem, Liderança, Biografia, História da Enfermagem

## Abstract

Trata-se de uma pesquisa histórica, com enfoque biográfico que utilizou o referencial foucaultiano como teoria subjacente para análise de dados.

**Objetivo:**

Analisar a trajetória profissional e política de Doris Grinspun, evidenciando suas contribuições para a enfermagem canadense e internacional à luz dos conceitos foucaultianos de poder, saber e subjetivação.

**Método:**

Pesquisa histórica com abordagem qualitativa, com enfoque biográfico. Os dados foram produzidos por entrevista semiestruturada, realizada em ambiente virtual, e submetidos à análise temática de conteúdo. A interpretação fundamentou-se na teoria subjacente dos conceitos de tecnologias de si, poder-saber, governamentalidade e regimes de verdade, propostos por Michel Foucault. O estudo atendeu aos critérios do Consolidated Criteria for Reporting Qualitative Research e aos preceitos éticos vigentes.

**Resultados:**

A trajetória evidencia processos de subjetivação constituídos desde a infância, mediados por experiências familiares, acadêmicas e profissionais, que favoreceram a construção de uma liderança ética e política. Destacam-se o engajamento sociopolítico, a articulação entre prática, formação e evidências e a atuação estratégica em organizações profissionais, com repercussão internacional.

**Conclusão:**

Sua liderança configura fenômeno ético-político que fortalece a governamentalidade e a visibilidade global da enfermagem.

## INTRODUÇÃO

A enfermagem, enquanto prática social e profissional, constitui-se historicamente como um espaço de disputas simbólicas e materiais atravessado por relações de poder, saber e identidade. Na perspectiva de Michel Foucault, os sujeitos não são entidades autônomas isoladas, mas emergem em meio a dispositivos de poder e técnicas de si que configuram modos de ser e agir inscritos no tecido social^(1,2)^. Compreender trajetórias de lideranças em enfermagem, portanto, ultrapassa a análise biográfica individual: exige reconhecer processos históricos, discursivos e institucionais que moldam subjetividades e reconfiguram racionalidades profissionais.

Pesquisas sobre enfermeiras militantes demonstram que o engajamento político-profissional surge da tensão entre alienação e emancipação e entre subalternidade e protagonismo^(3)^. A enfermagem, marcada por invisibilidades históricas e por vínculos com modelos biomédicos e religiosos, tem na liderança institucional e na militância caminhos para afirmar sua identidade coletiva e ampliar sua relevância social^(4)^. Nessas trajetórias, articulam-se práticas discursivas, processos educativos e racionalidades institucionais que configuram modos específicos de subjetivação, favorecendo a emergência de lideranças capazes de disputar sentidos da profissão e influenciar transformações estruturantes^(5)^.

Nesse cenário, a trajetória de Doris Grinspun no contexto canadense revela-se singular. Sua atuação transcende a prática clínica e configura um projeto ético-político de fortalecimento da enfermagem como profissão crítica, autônoma e comprometida com a justiça social. Ao liderar debates sobre políticas públicas, formação profissional, qualidade assistencial e representação institucional, Grinspun exemplifica o sujeito político foucaultiano: aquele que age sobre os outros, estabelece relações de poder e, simultaneamente, se constitui por meio de práticas de liberdade. Sua biografia explicita a articulação entre tecnologias de si e estratégias de governamentalidade que possibilitam reorientar instituições, discursos e práticas da enfermagem em escala nacional e internacional.

Doris Grinspun nasceu no Chile, em 1955, em uma família de raízes judaicas que posteriormente emigrou para Israel, onde ela iniciou sua formação acadêmica. Em sua vida pessoal, Doris é casada com o Dr. Elliot Grinspun e o casal possui filhos, mantendo uma base familiar sólida que a acompanhou durante sua transição e consolidação profissional no Canadá a partir da década de 1980.

A história de vida de Doris Grinspun vincula a sua figura a uma estrategista de saúde global. Sua maior contribuição é o movimento para que a enfermagem tome decisões baseadas nas melhores evidências científicas disponíveis, elevando o status da profissão e a qualidade do cuidado ao paciente.

Examinar sua história de vida permite situar a enfermagem canadense em um horizonte mais amplo de lutas políticas e profissionais, articulando formação, engajamento associativo e enfrentamento de modelos hegemônicos de cuidado^(6,7)^. Trata-se de compreender que a trajetória de Grinspun não é narrativa individual, mas expressão de processos sociais e profissionais que buscam superar a alienação, consolidar identidades coletivas e afirmar a enfermagem como prática social eticamente orientada. Essa perspectiva foucaultiana ilumina como se produzem regimes de verdade, racionalidades político-profissionais e subjetividades que sustentam transformações no campo da saúde^(8)^.

Assim, o objetivo deste artigo é analisar a trajetória de Doris Grinspun, destacando suas contribuições para a enfermagem canadense e internacional e situando sua militância e liderança como elementos centrais nos processos de transformação da profissão. Orienta a análise a seguinte questão: De que modo a trajetória de vida e a militância política de Doris Grinspun contribuíram para a constituição da enfermagem canadense como prática social, ética e cidadã, e quais dispositivos de poder e saber, à luz do referencial foucaultiano, se evidenciam em sua atuação profissional e institucional? Espera-se contribuir para o debate científico sobre identidade, política e poder, oferecendo elementos teórico-analíticos para compreender o papel das lideranças contemporâneas na consolidação da enfermagem como campo de saber, prática e intervenção social.

## MÉTODO

### Tipo de Estudo

Trata-se de uma pesquisa histórica com abordagem qualitativa, com enfoque biográfico da trajetória profissional da enfermeira Doris Grinspun. No âmbito da pesquisa qualitativa, esse tipo de abordagem permite explorar, descobrir e avaliar como as pessoas, através de suas experiências, compreendem seu passado, o vinculam ao contexto social e o significam no momento presente^(9)^. Estas possibilidades metodológicas convergem entre si: o referencial teórico-filosófico de Foucault, a pesquisa histórica e o enfoque biográfico são perspectivas teóricas que agregam maior profundidade às pesquisas qualitativas, sendo que estas têm como propósito a análise crítica e robusta dos dados e não a capacidade de generalização dos resultados.

Nesta direção, para compreensão da biografia em questão, foi utilizado o referencial filosófico foucaultiano, que aborda a constituição de sujeitos e aponta para os aspectos teóricos das técnicas de si, divididas em quatro grupos principais: (1) técnicas de produção, que permitem produzir, transformar ou manipular as coisas; (2) técnicas dos sistemas de signos, que permitem utilizar signos, sentidos, símbolos ou significação; (3) técnicas de poder, que determinam a conduta dos indivíduos e os submetem a certos fins ou dominação, objetivando o sujeito; (4) técnicas de si, que permitem aos indivíduos efetuar, com seus próprios meios ou com a ajuda de outros, um certo número de operações em seus próprios corpos, almas, pensamentos, conduta e modo de ser, de modo a transformá-los com o objetivo de alcançar um estado de felicidade, pureza, sabedoria, perfeição ou imortalidade^(2)^.

### População

No intuito de realizar uma reconstrução histórica da biografada, foi utilizada como técnica de coleta de dados a entrevista semiestruturada, conduzida em espanhol, a língua materna da entrevistada para a produção de fonte oral.

### Coleta de Dados

A entrevista ocorreu de forma virtual, por meio da plataforma online *Google Meet*. A coleta de dados foi realizada entre agosto e setembro de 2021, com duração aproximada de 1 hora e 20 minutos, garantindo condições adequadas de registro e preservação da narrativa.

### Análise e Tratamento dos Dados

Para a análise dos dados, optou-se pelo método de análise temática de conteúdo, que contemplou as seguintes etapas: recorte das unidades de registro, enumeração com base na frequência, tratamento dos resultados e interpretações. Válido destacar que esta opção agrega na possibilidade de uma análise de dados com maior profundidade mediada por uma teoria subjacente que versa sobre a análise do sujeito. A categorização dos dados a partir de um referencial teórico-filosófico sustentou-se nas unidades de registro e no conteúdo manifesto. A análise não se limitou à frequência das unidades de gravação, mas priorizou a interpretação de práticas discursivas, relações de poder e processos de subjetivação.

O posicionamento analítico partiu da obra de Foucault, com categorias pré-estabelecidas, mas dando espaço para as idiossincrasias que apareceriam no decorrer da análise de dados. As categorias emergentes foram posteriormente confrontadas com o referencial foucaultiano, em uma estrutura analítica que permitiu identificar dispositivos de poder, técnicas de si e modos de constituição de sujeitos. Na etapa de organização dos dados, foi utilizado como apoio o *software* de Análise Qualitativa de Dados WebQDA®, que possibilitou sistematizar e explorar em profundidade os conteúdos da entrevista^(10)^.

### Aspectos Éticos

Esta pesquisa integra o projeto matriz intitulado “Modelos de Gestão em Enfermagem: memórias de enfermeiras”, aprovado no Comitê de Ética em Pesquisa sob Parecer nº 5.462.599, sendo todas as normativas de pesquisa com seres humanos respeitadas. A entrevista só ocorreu mediante autorização da entrevistada e assinatura do termo de concessão dos direitos autorais, sendo posteriormente transcrita e validada pela própria participante antes de sua utilização para análise^(11)^. As falas oriundas da entrevista estão identificadas pelas letras “ENT”, seguidas pelas iniciais do nome da entrevistada “DG”.

Todo o processo foi guiado pelo *Consolidated Criteria for Reporting Qualitative Research* (COREQ), versão em português, que estabelece critérios para relatar pesquisas qualitativas, assegurando transparência, rigor e reprodutibilidade^(12)^.

Houve utilização focada de inteligência artificial para o auxílio à escrita e estruturação do texto.

## RESULTADOS

As narrativas evidenciam uma trajetória permeada por práticas precoces de organização coletiva, as quais delineiam modos de subjetivação atrelados ao exercício da liderança. Desde a infância, a biografada descreve a constituição de agrupamentos estruturados, dotados de normas e dispositivos de pertencimento, o que sinaliza uma orientação precoce à coordenação de pares e à gestão do comum.

Os discursos apresentados no Quadro 1 situam essas experiências em três eixos: a análise do discurso, das práticas e dos projetos na formação de enfermeiras. Com base nesses relatos, o Quadro 2 reúne as categorias analíticas e explicita os processos foucaultianos mobilizados na interpretação da trajetória da biografada.

**Quadro 1:** O discurso, as práticas e os projetos de Doris Grinspun – Salvador, BA, Brasil, 2026.

A análise do discurso na formação de enfermeiras	
O discurso das experiências de liderança	*Yo formé el club de las pulguitas – risas – (…) teníamos carnet que los plastificamos con las velitas. (ENT-DG)*
	*Ellas formaron su grupo… y yo dije ‘¿Y el resto de nosotras qué vamos a hacer?’. Club de la pulguita, y obviamente si lo vamos a hacer, lo vamos a hacer mejor. (ENT-DG)*
	*Después me hice líder en el movimiento social (…) fui a Uruguay a hacer un curso de líder de juventud y me transformé en una de las líderes del movimiento Barclays. (ENT-DG)*
	*Siempre en cada país que estuve empecé como enfermera clínica porque quería entender la práctica del país (…) y lo segundo que hice fue obtener un título académico en cada país. (ENT-DG)*
O discurso das experiências familiares	*Desde los 6 años tenía que decir algo sobre lo que el diario decía en la comida… y los viernes teníamos que cantar. (ENT-DG)*
	*Nos educaron a ser libres… mi padre quería que sus hijas vuelen. (ENT-DG)*
	*Imagínate… estaba al otro lado del mundo, en un país de guerra, con un idioma que no conocía bien, y mis padres solo decían ‘que te vaya bien’. (ENT-DG)*
O discurso do processo acadêmico	*Una cosa es saber el idioma y otra cosa es hablarlo… cuando llegué todo era una línea y yo no entendía nada. (ENT-DG)*
	*Te hicieron buena enfermera, muy disciplinada, pero había cosas absurdas… te hacían separar tus problemas de los pacientes. (ENT-DG)*
	*Me dijeron que abra una unidad para soldados con lesiones… yo dije ‘soy estudiante’, pero respondieron ‘dijeron que tú podías hacerlo’. Me miré al espejo y dije ‘si ellos dicen que puedo, será porque puedo’. (ENT-DG)*
A análise das práticas na formação de enfermeiras	
Práticas do trabalho da enfermeira	*Si al centro de tu atención están los pacientes, nunca vas a hacer algo mal. (ENT-DG)*
	*Revolucioné todo el hospital sin saber que lo estaba haciendo, porque pensaba primero en los pacientes. (ENT-DG)*
	*Yo vine a la profesión porque me enamoré de la gente… la enfermería es una herramienta para ayudar al prójimo. (ENT-DG)*
	*Lo que dije fue: gracias, gracias por haber trabajado… y por favor quédense, las crisis no duran para siempre. (ENT-DG)*
A prática da enfermagem durante a pandemia	*La pandemia nos dio en bandeja la oportunidad de lucirnos como profesión… pero no hubo un poder coordinador internacional. (ENT-DG)*
	*Si se nos hubiera escuchado, estaríamos mil veces mejor. (ENT-DG)*
As práticas de engajamento sociopolítico	*Trabajar en la guerra me hizo pacifista y activista de la paz… eso no se olvida nunca. (ENT-DG)*
	*Buscaba la justicia humana… siempre fue mi intención cambiar el mundo. (ENT-DG)*
	*El poder no sirve para nada si no se comparte… mientras más lo compartes, mejor para todos. (ENT-DG)*
A análise dos projetos na formação de enfermeiras	
Enfermagem: perspectivas sobre presente e futuro	*Si los estudiantes salen con la expectativa de practicar basado en la evidencia, ganamos. (ENT-DG)*
	*La mejor oportunidad es investigación centrada en la práctica, no artículos por artículos. (ENT-DG)*
Cenário das boas práticas em enfermagem	*Es un movimiento social de conocimiento. (ENT-DG)*
	*Cuando llegué teníamos 9.000 miembros… ahora somos 46.000. (ENT-DG)*
	*Nosotros damos metodología y materiales; ellos nos dan cultura, liderazgo y riqueza humana… aprendemos juntos. (ENT-DG)*
Impacto do programa na vida das pessoas	*Cuando veo emails de gente que ni conozco que me dicen la diferencia que hice en sus vidas… eso es lo más importante. (ENT-DG)*

Fonte: Elaborado pelos autores, 2026.

**Quadro 2:** Síntese das categorias analíticas – Salvador, BA, Brasil, 2026.

Categoria analítica	Conteúdos centrais	Excerto(s) relevante(s)	Processos foucaultianos mobilizados	Síntese interpretativa
Experiências de liderança	Formação precoce, organização entre pares, cursos de liderança juvenil	*“Yo formé el club de las pulguitas…”*	Dispositivos de governamentalidade; técnicas de si	Liderança emerge de práticas precoces de organização e reconhecimento
Experiências familiares	Autonomia, debate crítico, educação emancipatória	*“nos empujaban la máquina del pensar y del cuestionar”*	Tecnologias de si	Família como espaço formador de subjetividade crítica e ética
Processo acadêmico e disciplina	Barreiras linguísticas, formação rigorosa, deslocamentos culturais	*“te hicieron buena enfermera… disciplinados pero absurdos”*	Disciplinamento; regimes de verdade	Formação molda corpo profissional por disciplina e resistência
Prática clínica e ética do cuidado	Cuidado centrado no paciente, impacto institucional	*“si al centro de tu atención están los pacientes…”*	Poder-saber; governamento do cuidado	Cuidado fundamenta liderança ética e transformadora
Pandemia e disputas políticas	Falta de escuta, crise global, potencial não aproveitado	*“si se nos hubiera escuchado…”*	Disputas discursivas; biopolítica	Pandemia revela tensões entre visibilidade e limitação política
Engajamento sociopolítico	Ativismo pela paz, justiça humana	*“me hizo pacifista y activista de la paz”*	Práticas de liberdade; resistência	Engajamento político orienta liderança ética e coletiva
Projetos para o futuro	Formação baseada em evidências, integração prática-pesquisa	*“investigación centrada en la práctica”*	Produção de saberes; governamentalidade	Futuro depende de articulação entre prática, pesquisa e ética
Boas práticas e expansão	Programa internacional, coaprendizagem, impacto em pacientes	*“es un movimiento social de conocimiento”*	Redes de poder-saber; internacionalização	Programa como dispositivo global de fortalecimento da enfermagem

Fonte: Elaborado pelos autores, 2026.

## DISCUSSÃO

A trajetória profissional e acadêmica de Doris Grinspun manifesta um percurso singular em que experiências de vida, investimento educacional, práticas clínicas e atuação institucional se constituem mutuamente. Para compreendê-la, é necessário analisá-la como produto de relações históricas, discursivas e políticas que atravessam a enfermagem contemporânea. Nesta perspectiva, adotamos como eixo interpretativo o referencial foucaultiano, que permite compreender a formação da liderança de Grinspun como processo de subjetivação e sua atuação política e institucional como expressão de técnicas de poder e de governamentalidade. Adotamos aqui a leitura foucaultiana como enquadre analítico que integra evidências empíricas – segmentos discursivos da entrevista – e categorias conceituais (tecnologias disciplinares, tecnologias do eu, regimes de verdade, poder-saber, governamentalidade), de modo a explicitar como práticas e discursos moldam sujeitos e práticas profissionais. Organiza-se a discussão em quatro blocos analíticos: (1) impacto do contexto familiar e da socialização profissional; (2) transformação da formação em enfermagem na América Latina; (3) inovações em pesquisa, currículo e simulação; e (4) liderança institucional e advocacy – governamentalidade, resistência e expansão da profissão.

### Impacto do Contexto Familiar, Socialização e Produção de Sujeitos em Enfermagem

A trajetória inicial de Grinspun indica que valores familiares, mobilidade transnacional, experiências de enfrentamento e incentivo à educação constituíram dispositivos elementares de subjetivação. Segundo Foucault^(1)^, o sujeito não é uma origem autônoma, mas o efeito de práticas, discursos e relações de poder que modelam modos de ser, agir e pensar^(2)^. Assim, as vivências relatadas por Grinspun – liderança precoce, consciência social, exposição a contextos de conflito e educação crítica – funcionam como matrizes discursivas que produziram uma profissional orientada à ética, à responsabilidade coletiva e ao engajamento político. Mais precisamente, essas práticas familiares e formativas operam como “tecnologias do eu” (technologies of the self): procedimentos pelos quais o indivíduo se constitui, através de exercícios de julgamento, disciplina e cuidado de si, internalizando normas e desenvolvendo capacidades de ação política e ética.

A literatura sobre socialização profissional reafirma que experiências na infância, deslocamentos geográficos e situações de vulnerabilidade influenciam a constituição de identidades profissionais comprometidas com a justiça social^(13,14)^. Autores apontam que trajetórias biográficas frequentemente articulam práticas educativas familiares e formação moral – dimensão explicitamente presente no relato de Grinspun^(15)^. Do ponto de vista foucaultiano, a infância e a juventude aqui não são meros antecedentes temporais, mas dispositivos históricos que inscrevem modos de subjetivação: produzem predisposições para a crítica, para o exercício responsivo da autonomia e para a ação coletiva. Assim, a constituição de Grinspun como sujeito político é antecipada por uma rede de práticas que articulam liberdade, disciplina e compromisso ético, e que posteriormente serão mobilizadas no âmbito institucional e discursivo da enfermagem.

### Transformação e a Formação em Enfermagem no Brasil e na América Latina

A análise foucaultiana ilumina processos macroestruturais que atravessam a formação profissional. Na América Latina, o campo formativo é tensionado por desigualdades, fragmentação curricular e heterogeneidade de padrões^(16)^. A atuação de Grinspun insere-se nesse contexto como força que busca deslocar racionalidades hegemônicas de ensino, aproximando formação e prática clínica com base em evidências internacionais^(17)^. Esses deslocamentos configuram operações de governamentalidade educacional: enunciam normas, métricas e procedimentos que orientam condutas de estudantes, docentes e gestores, ao mesmo tempo em que produzem sujeitos governáveis (profissionais formados segundo critérios de eficiência e segurança).

A defesa de uma educação qualificada, integrada e centrada em competências dialoga com o debate nacional sobre a articulação ensino-serviço e a necessidade de currículos críticos e reflexivos^(18)^. No horizonte foucaultiano, a difusão dessas racionalidades implica a constituição de regimes de verdade educacionais – conjuntos de enunciados e práticas que definem o que é considerado ‘*conhecimento válido*’ e ‘*boa prática*’ na formação. Assim, a influência de Grinspun transcende a mera melhoria técnica de conteúdos: ela reconfigura o regime discursivo que determina o que deve ser ensinado, como deve ser avaliado e que tipo de profissional se pretende formar.

### Inovações em Pesquisa, Currículo e Simulação: Práticas de Saber e Produção de Verdade

As inovações promovidas por Grinspun – desenvolvimento curricular, investigação aplicada e uso de simulação realística – articulam práticas de saber e regimes de verdade que estruturam a enfermagem contemporânea. Para Foucault, discursos científicos são mecanismos centrais na produção de verdade, capazes de legitimar práticas e orientar condutas. Nesse sentido, a simulação e a pesquisa centrada na prática não apenas transmitem competências técnicas; constituem espaços onde se experimentam, validam e difundem enunciados de verdade sobre o cuidado, produzindo sujeitos que incorporam tais saberes em suas práticas cotidianas.

A criação e implementação das Best Practice Guidelines (BPGs) representam um exemplo paradigmático de dispositivo de poder-saber: as BPGs articulam ciência, política e prática ao traduzir evidências em padrões e recomendações que padronizam condutas, reduzem variabilidade assistencial e orientam decisões clínicas. Sob o olhar foucaultiano, trata-se de tecnologias que disciplinam práticas ao mesmo tempo em que podem promover autonomia profissional – porque proporcionam um arcabouço técnico que capacita juízo clínico informado por evidência. Dessa forma, as BPGs desempenham um duplo papel: disciplinam por meio de normativas e instrumentos de controle, e emancipam ao oferecer recursos para a tomada de decisão baseada em critérios técnicos e éticos. Esse caráter ambivalente merece atenção analítica: nem meramente opressivas nem puramente libertadoras, as práticas-guia inscrevem possibilidades de agência dentro de limites institucionais.

### Liderança Institucional e Advocacy: Governamentalidade, Resistência e Expansão da Profissão

Na dimensão institucional, Grinspun demonstra uma liderança que visa transformar estruturas e práticas. Sua atuação na *Registered Nurses’ Association of Ontario* (RNAO) exemplifica a capacidade de articular políticas, mobilizar coletivos e influenciar agendas públicas – práticas que podem ser enquadradas como formas de governamentalidade profissional. Segundo Foucault, governamentalidade refere-se às técnicas e racionalidades com as quais se conduz o comportamento de populações e instituições; nesse sentido, a construção de programas, redes e estratégias de advocacy pela RNAO constitui um processo de condução das condutas profissionais e da política de saúde.

A literatura indica que a profissionalização exige lideranças capazes de disputar sentidos da prática e promover justiça social^(19,20)^. Grinspun atua neste campo fortalecendo redes e ampliando a visibilidade da enfermagem em arenas políticas, como evidenciado durante a pandemia e nas campanhas por justiça na distribuição de vacinas. Sua ação ilustra também o conceito foucaultiano de resistência: não como mera negação do poder, mas como produção de alternativas – de formas distintas de organização, conhecimento e intervenção política que reconfiguram o espaço das possibilidades institucionais.

A afirmação de Grinspun de que as experiências de guerra a converteram em “*ativista da paz*” pode ser lida como prática de constituição ética do sujeito: um exercício de tecnologia do eu que orienta escolhas públicas e modos de engajamento coletivo.Assim, sua trajetória institucional opera simultaneamente como produção de saber, exercício de poder e produção de subjetividade – um nó que exemplifica como liderança, intervenção política e produção de conhecimento se entrelaçam para ampliar a governamentalidade da enfermagem.

Nessa perspectiva, a análise foucaultiana da trajetória de Doris Grinspun revela que sua potência como liderança advém da articulação entre processos de subjetivação (tecnologias do eu), dispositivos disciplinares (protocolos, currículos, BPGs) e estratégias de governamentalidade (ações institucionais e advocacy). Essa articulação mostra que o poder, na enfermagem contemporânea, não se exerce apenas como coerção, mas como produção: de saberes, de modos de conduta e de sujeitos políticos capazes de intervir nas estruturas que os produziram.

Portanto, a biografia de Grinspun constitui um material heurístico para compreender como agentes profissionais podem instituir deslocamentos discursivos e institucionais que ampliam margens de ação, redefinem regimes de verdade e promovem mudanças na formação e nas políticas de saúde. Do ponto de vista prático, essa leitura sugere que políticas formativas e de gestão devem reconhecer a dupla dinâmica dos dispositivos (disciplinar/emancipatório) e investir em estratégias que fomentem tanto a produção de evidências aplicáveis quanto o desenvolvimento de tecnologias do eu que sustentem a prática ética e crítica.

As limitações deste estudo decorrem de seu delineamento histórico-biográfico centrado na narrativa de uma única liderança, produzida por entrevista virtual e interpretada à luz exclusiva do referencial foucaultiano, o que circunscreve a abrangência analítica, restringe a triangulação de fontes e limita a generalização dos achados para outros contextos sociopolíticos da enfermagem global.

## CONCLUSÃO

A análise foucaultiana da trajetória de Doris Grinspun revela que sua liderança transcende realizações individuais e se configura como dispositivo de transformação capaz de reorganizar modos de ser, agir e pensar a enfermagem em perspectiva global. Ao articular prática clínica, gestão de sistemas, pesquisa aplicada, inovação educacional e ativismo político, Grinspun opera no entrelaçamento entre poder e saber, contribuindo para reconfigurar formações, práticas e políticas de saúde.

Seu legado mostra que a enfermagem amplia sua governamentalidade quando ocupa posições estratégicas na formulação de políticas, na produção de evidências e na defesa de agendas orientadas ao bem comum. Essa atuação ilumina caminhos especialmente relevantes para países do Sul Global, evidenciando que a ampliação da autonomia profissional depende de lideranças capazes de intervir criticamente nas racionalidades hegemônicas que moldam os sistemas de saúde.

Ao destacar como dispositivos disciplinares, tecnologias de si e estratégias de governamentalidade se articulam na constituição de sua liderança, este estudo reafirma a biografia como ferramenta metodológica potente para compreender processos de mudança profissional. A trajetória de Doris Grinspun, marcada por compromisso ético, visão estratégica e engajamento político, configura-se como referência internacional para a construção de lideranças críticas, inovadoras e transformadoras. Evidencia-se, por fim, que liderança em enfermagem é fenômeno ético-político capaz de produzir novos regimes de verdade, transgredir fronteiras institucionais e projetar horizontes ampliados para a profissão em escala global.

Por fim, o estudo apresenta como limitação a ausência de triangulação dos resultados com outras fontes. Contudo, nota-se a necessidade de explicitar, com maior profundidade analítica, a história de vida da biografada a partir da entrevista. Tal abordagem configura-se como uma oportunidade para aplicar o referencial filosófico de Michel Foucault, explorando as técnicas de si na construção do sujeito.

## DISPONIBILIDADE DE DADOS

Todo o conjunto de dados que dá suporte aos resultados deste estudo foi publicado no próprio artigo.

# Translation: Liderazgo, poder y subjetivación en la enfermería global: la trayectoria de Doris Grinspun

**DOI:** 10.1590/1980-220X-REEUSP-2026-0192es

**Authors:** Amalia Graciela Silva Galleguillos, Oscar Javier Vergara Escobar, Ingredy Nayara Chiacchio Silva, Deybson Borba de Almeida, Gilberto Tadeu Reis da Silva

**Affiliations:** 1 Universidad de Chile, Santiago, Chile.; 2 Universidad El Bosque, Bogotá, Colombia.; 3 Universidade Federal da Bahia, Salvador, BA, Brasil.; 4 Universidade Estadual de Feira de Santana, Feira de Santana, BA, Brasil.

**Keywords:** Enfermería, Liderazgo, Biografía, Historia de la enfermería

## Abstract

Se trata de una investigación histórica, con enfoque biográfico, que utilizó el marco foucaultiano como teoría subyacente para el análisis de datos.

**Objetivo:**

Analizar la trayectoria profesional y política de Doris Grinspun, poniendo de relieve sus contribuciones a la enfermería canadiense e internacional a la luz de los conceptos foucaultianos de poder, saber y subjetivación.

**Método:**

Investigación histórica con enfoque cualitativo y enfoque biográfico. Los datos se obtuvieron mediante una entrevista semiestructurada, realizada en un entorno virtual, y se sometieron a un análisis temático de contenido. La interpretación se basó en la teoría subyacente de los conceptos de tecnologías del yo, poder-saber, gubernamentalidad y regímenes de verdad, propuestos por Michel Foucault. El estudio cumplió con los criterios de los Criterios Consolidados para el Informe de Investigación Cualitativa y con los preceptos éticos vigentes.

**Resultados:**

La trayectoria pone de manifiesto procesos de subjetivación constituidos desde la infancia, mediados por experiencias familiares, académicas y profesionales, que favorecieron la construcción de un liderazgo ético y político. Destacan el compromiso sociopolítico, la articulación entre la práctica, la formación y la evidencia, y la actuación estratégica en organizaciones profesionales, con repercusión internacional.

**Conclusión:**

Su liderazgo constituye un fenómeno ético-político que fortalece la gubernamentalidad y la visibilidad global de la enfermería.

## INTRODUCCIÓN

La enfermería, como práctica social y profesional, se ha constituido históricamente como un espacio de disputas simbólicas y materiales atravesado por relaciones de poder, conocimiento e identidad. Desde la perspectiva de Michel Foucault, los sujetos no son entidades autónomas aisladas, sino que surgen en medio de dispositivos de poder y técnicas del yo que configuran modos de ser y actuar inscritos en el tejido social^(1,2)^. Comprender las trayectorias de las figuras de liderazgo en enfermería, por lo tanto, va más allá del análisis biográfico individual: exige reconocer los procesos históricos, discursivos e institucionales que moldean las subjetividades y reconfiguran las racionalidades profesionales.

Las investigaciones sobre enfermeras militantes demuestran que el compromiso político-profesional surge de la tensión entre la alienación y la emancipación, y entre la subalternidad y el protagonismo^(3)^. La enfermería, marcada por invisibilidades históricas y por vínculos con modelos biomédicos y religiosos, encuentra en el liderazgo institucional y en la militancia vías para afirmar su identidad colectiva y ampliar su relevancia social^(4)^. En estas trayectorias se articulan prácticas discursivas, procesos educativos y racionalidades institucionales que configuran modos específicos de subjetivación, favoreciendo el surgimiento de lideranzas capaces de disputar los significados de la profesión e influir en transformaciones estructurales^(5)^.

En este escenario, la trayectoria de Doris Grinspun en el contexto canadiense resulta singular. Su labor trasciende la práctica clínica y configura un proyecto ético-político de fortalecimiento de la enfermería como profesión crítica, autónoma y comprometida con la justicia social. Al liderar debates sobre políticas públicas, formación profesional, calidad de la atención y representación institucional, Grinspun ejemplifica al sujeto político foucaultiano: aquel que actúa sobre los demás, establece relaciones de poder y, simultáneamente, se constituye a través de prácticas de libertad. Su biografía pone de manifiesto la articulación entre las tecnologías del yo y las estrategias de gubernamentalidad que permiten reorientar las instituciones, los discursos y las prácticas de la enfermería a escala nacional e internacional.

Doris Grinspun nació en Chile en 1955, en una familia de raíces judías que posteriormente emigró a Israel, donde ella comenzó su formación académica. En su vida personal, Doris está casada con el Dr. Elliot Grinspun y la pareja tiene hijos, lo que le ha permitido mantener una base familiar sólida que la acompañó durante su transición y consolidación profesional en Canadá a partir de la década de 1980.

La trayectoria de vida de Doris Grinspun la vincula con su faceta de estratega de la salud global. Su mayor contribución es el impulso para que la enfermería tome decisiones basadas en la mejor evidencia científica disponible, elevando así el estatus de la profesión y la calidad de la atención al paciente.

Al examinar su trayectoria de vida, se puede situar a la enfermería canadiense en un horizonte más amplio de luchas políticas y profesionales, que articula la formación, el compromiso asociativo y el desafío a los modelos hegemónicos de atención^(6,7)^. Se trata de comprender que la trayectoria de Grinspun no es una narrativa individual, sino la expresión de procesos sociales y profesionales que buscan superar la alienación, consolidar identidades colectivas y afirmar la enfermería como una práctica social orientada éticamente. Esta perspectiva foucaultiana ilumina cómo se producen los regímenes de verdad, las racionalidades político-profesionales y las subjetividades que sustentan las transformaciones en el campo de la salud^(8)^.

Así, el objetivo de este artículo es analizar la trayectoria de Doris Grinspun, destacando sus contribuciones a la enfermería canadiense e internacional y situando su activismo y liderazgo como elementos centrales en los procesos de transformación de la profesión. El análisis se orienta en torno a la siguiente pregunta: ¿De qué manera la trayectoria de vida y el activismo político de Doris Grinspun contribuyeron a la constitución de la enfermería canadiense como práctica social, ética y ciudadana, y qué dispositivos de poder y saber, a la luz del marco foucaultiano, se evidencian en su actuación profesional e institucional? Se espera contribuir al debate científico sobre identidad, política y poder, ofreciendo elementos teórico-analíticos para comprender el papel de los líderes contemporáneos en la consolidación de la enfermería como campo de conocimiento, práctica e intervención social.

## MÉTODO

### Tipo de Estudio

Se trata de una investigación histórica con enfoque cualitativo, que adopta una perspectiva biográfica de la trayectoria profesional de la enfermera Doris Grinspun. En el ámbito de la investigación cualitativa, este tipo de enfoque permite explorar, descubrir y evaluar cómo las personas, a través de sus experiencias, comprenden su pasado, lo vinculan al contexto social y le dan sentido en el momento presente^(9)^. Estas posibilidades metodológicas convergen entre sí: el marco teórico- filosófico de Foucault, la investigación histórica y el enfoque biográfico son perspectivas teóricas que aportan mayor profundidad a las investigaciones cualitativas, las cuales tienen como propósito el análisis crítico y sólido de los datos y no la capacidad de generalización de los resultados.

En este sentido, para comprender la biografía en cuestión, se utilizó el marco filosófico foucaultiano, que aborda la constitución de los sujetos y señala los aspectos teóricos de las técnicas del yo, divididas en cuatro grupos principales: (1) técnicas de producción, que permiten producir, transformar o manipular las cosas; (2) técnicas de los sistemas de signos, que permiten utilizar signos, sentidos, símbolos o significados; (3) técnicas de poder, que determinan la conducta de los individuos y los someten a ciertos fines o a la dominación, objetivando al sujeto; (4) técnicas del yo, que permiten a los individuos realizar, con sus propios medios o con la ayuda de otros, una serie de operaciones en sus propios cuerpos, almas, pensamientos, conducta y forma de ser, con el fin de transformarlos para alcanzar un estado de felicidad, pureza, sabiduría, perfección o inmortalidad^(2)^.

### Población

Con el fin de llevar a cabo una reconstrucción histórica de la biografiada, se utilizó como técnica de recolección de datos la entrevista semiestructurada, realizada en español, la lengua materna de la entrevistada, para la producción de la fuente oral.

### Recolección de Datos

La entrevista se llevó a cabo de manera virtual, a través de la plataforma en línea *Google Meet*. La recolección de datos se realizó entre agosto y septiembre de 2021, con una duración aproximada de 1 hora y 20 minutos, garantizando condiciones adecuadas para el registro y la preservación de la narrativa.

### Análisis y Tratamiento de los Datos

Para el análisis de los datos, se optó por el método de análisis temático de contenido, que incluyó las siguientes etapas: extracción de las unidades de registro, recuento basado en la frecuencia, tratamiento de los resultados e interpretaciones. Cabe destacar que esta opción ofrece la posibilidad de un análisis de datos más profundo, mediado por una teoría subyacente que aborda el análisis del sujeto. La categorización de los datos a partir de un marco teórico-filosófico se basó en las unidades de registro y en el contenido manifiesto. El análisis no se limitó a la frecuencia de las unidades de registro, sino que priorizó la interpretación de las prácticas discursivas, las relaciones de poder y los procesos de subjetivación.

El enfoque analítico partió de la obra de Foucault, con categorías preestablecidas, pero dejando espacio para las idiosincrasias que surgieran en el transcurso del análisis de los datos.

Las categorías emergentes se confrontaron posteriormente con el marco foucaultiano, en una estructura analítica que permitió identificar dispositivos de poder, técnicas del yo y modos de constitución de sujetos. En la etapa de organización de los datos, se utilizó como apoyo el *software* de Análisis Cualitativo de Datos WebQDA®, lo que permitió sistematizar y explorar en profundidad los contenidos de la entrevista^(10)^.

### Aspectos Éticos

Esta investigación forma parte del proyecto principal titulado “Modelos de gestión en enfermería: memorias de enfermeras”, aprobado por el Comité de Ética en Investigación mediante el Dictamen n.º 5.462.599, habiéndose respetado todas las normas relativas a la investigación con seres humanos. La entrevista solo se llevó a cabo con la autorización de la entrevistada y la firma del documento de cesión de derechos de autor, y posteriormente fue transcrita y validada por la propia participante antes de su uso para el análisis^(11)^. Las declaraciones procedentes de la entrevista están identificadas con las letras “ENT”, seguidas de las iniciales del nombre de la entrevistada “DG”.

Todo el proceso se guió por los *Consolidated Criteria for Reporting Qualitative Research* (COREQ), versión en portugués, que establecen criterios para informar sobre investigaciones cualitativas, garantizando transparencia, rigor y reproducibilidad^(12)^.

Se hizo un uso específico de la inteligencia artificial para facilitar la redacción y la estructuración del texto.

## RESULTADOS

Las narrativas evidencian una trayectoria marcada por prácticas tempranas de organización colectiva, las cuales delinean modos de subjetivación vinculados al ejercicio del liderazgo. Desde la infancia, la biografiada describe la formación de agrupaciones estructuradas, dotadas de normas y mecanismos de pertenencia, lo que indica una orientación temprana hacia la coordinación entre pares y la gestión de lo común.

Los discursos presentados en el Cuadro 1 sitúan estas experiencias en tres ejes: el análisis del discurso, de las prácticas y de los proyectos en la formación de enfermeras. A partir de estos relatos, el Cuadro 2 reúne las categorías analíticas y explicita los procesos foucaultianos movilizados en la interpretación de la trayectoria de la biografiada.

**Cuadro 1:** El discurso, las prácticas y los proyectos de Doris Grinspun – Salvador, BA, Brasil, 2026.

El análisis del discurso en la formación de enfermeras	
El discurso de las experiencias de liderazgo	*Yo formé el club de las pulguitas – risas – (…) teníamos carnet que los plastificamos con las velitas. (ENT-DG)*
	*Ellas formaron su grupo… y yo dije ‘¿Y el resto de nosotras qué vamos a hacer?’. Club de la pulguita, y obviamente si lo vamos a hacer, lo vamos a hacer mejor. (ENT-DG)*
	*Después me hice líder en el movimiento social (…) fui a Uruguay a hacer un curso de líder de juventud y me transformé en una de las líderes del movimiento Barclays. (ENT-DG)*
	*Siempre en cada país que estuve empecé como enfermera clínica porque quería entender la práctica del país (…) y lo segundo que hice fue obtener un título académico en cada país. (ENT-DG)*
El discurso de las experiencias familiares	*Desde los 6 años tenía que decir algo sobre lo que el diario decía en la comida… y los viernes teníamos que cantar. (ENT-DG)*
	*Nos educaron a ser libres… mi padre quería que sus hijas vuelen. (ENT-DG)*
	*Imagínate… estaba al otro lado del mundo, en un país de guerra, con un idioma que no conocía bien, y mis padres solo decían ‘que te vaya bien’. (ENT-DG)*
El discurso del proceso académico	*Una cosa es saber el idioma y otra cosa es hablarlo… cuando llegué todo era una línea y yo no entendía nada. (ENT-DG)*
	*Te hicieron buena enfermera, muy disciplinada, pero había cosas absurdas… te hacían separar tus problemas de los pacientes. (ENT-DG)*
	*Me dijeron que abra una unidad para soldados con lesiones… yo dije ‘soy estudiante’, pero respondieron ‘dijeron que tú podías hacerlo’. Me miré al espejo y dije ‘si ellos dicen que puedo, será porque puedo’. (ENT-DG)*
El análisis de las prácticas en la formación de enfermeras	
Prácticas de trabajo de enfermería	*Si al centro de tu atención están los pacientes, nunca vas a hacer algo mal. (ENT-DG)*
	*Revolucioné todo el hospital sin saber que lo estaba haciendo, porque pensaba primero en los pacientes. (ENT-DG)*
	*Yo vine a la profesión porque me enamoré de la gente… la enfermería es una herramienta para ayudar al prójimo. (ENT-DG)*
	*Lo que dije fue: gracias, gracias por haber trabajado… y por favor quédense, las crisis no duran para siempre. (ENT-DG)*
La práctica de la enfermería durante la pandemia	*La pandemia nos dio en bandeja la oportunidad de lucirnos como profesión… pero no hubo un poder coordinador internacional. (ENT-DG)*
	*Si se nos hubiera escuchado, estaríamos mil veces mejor. (ENT-DG)*
Las prácticas de compromiso sociopolítico	*Trabajar en la guerra me hizo pacifista y activista de la paz… eso no se olvida nunca. (ENT-DG)*
	*Buscaba la justicia humana… siempre fue mi intención cambiar el mundo. (ENT-DG)*
	*El poder no sirve para nada si no se comparte… mientras más lo compartes, mejor para todos. (ENT-DG)*
El análisis de los proyectos en la formación de enfermeras	
Enfermería: perspectivas sobre el presente y el futuro	*Si los estudiantes salen con la expectativa de practicar basado en la evidencia, ganamos. (ENT-DG)*
	*La mejor oportunidad es investigación centrada en la práctica, no artículos por artículos. (ENT-DG)*
Panorama de las buenas prácticas en enfermería	*Es un movimiento social de conocimiento. (ENT-DG)*
	*Cuando llegué teníamos 9.000 miembros… ahora somos 46.000. (ENT-DG)*
	*Nosotros damos metodología y materiales; ellos nos dan cultura, liderazgo y riqueza humana… aprendemos juntos. (ENT-DG)*
Impacto del programa en la vida de las personas	*Cuando veo emails de gente que ni conozco que me dicen la diferencia que hice en sus vidas… eso es lo más importante. (ENT-DG)*

Fuente: Elaborado por los autores, 2026.

**Cuadro 2:** Resumen de las categorías analíticas – Salvador, BA, Brasil, 2026.

Categoría analítica	Contenidos centrales	Fragmento(s) relevante(s)	Procesos foucaultianos movilizados	Síntesis interpretativa
Experiencias de liderazgo	Formación temprana, organización entre pares, cursos de liderazgo juvenil	*“Yo formé el club de las pulguitas…”*	Dispositivos de gubernamentalidad; técnicas del yo	El liderazgo surge de prácticas tempranas de organización y reconocimiento
Experiencias familiares	Autonomía, debate crítico, educación emancipadora	*“nos empujaban la máquina del pensar y del cuestionar”*	Tecnologías del yo	La familia como espacio formador de subjetividad crítica y ética
Proceso académico y disciplina	Barreras lingüísticas, formación rigurosa, desplazamientos culturales	*“te hicieron buena enfermera… disciplinados pero absurdos”*	Disciplinamiento; regímenes de verdad	La formación moldea el cuerpo profesional a través de la disciplina y la resistencia
Práctica clínica y ética del cuidado	Atención centrada en el paciente, impacto institucional	*“si al centro de tu atención están los pacientes…”*	Poder-saber; gobierno del cuidado	El cuidado sustenta el liderazgo ético y transformador
Pandemia y disputas políticas	Falta de escucha, crisis global, potencial no aprovechado	*“si se nos hubiera escuchado…”*	Disputas discursivas; biopolítica	La pandemia revela tensiones entre la visibilidad y las limitaciones políticas
Compromiso sociopolítico	Activismo por la paz, justicia humana	*“me hizo pacifista y activista de la paz”*	Prácticas de libertad; resistencia	El compromiso político orienta el liderazgo ético y colectivo
Proyectos para el futuro	Formación basada en la evidencia, integración entre la práctica y la investigación	*“investigación centrada en la práctica”*	Producción de conocimientos; gubernamentalidad	El futuro depende de la articulación entre la práctica, la investigación y la ética
Buenas prácticas y expansión	Programa internacional, co-aprendizaje, impacto en pacientes	*“es un movimiento social de conocimiento”*	Redes de poder-saber; internacionalización	El programa como dispositivo global para fortalecer la enfermería

## DISCUSIÓN

La trayectoria profesional y académica de Doris Grinspun refleja un recorrido singular en el que las experiencias de vida, la inversión en educación, las prácticas clínicas y la actuación institucional se construyen mutuamente. Para comprenderla, es necesario analizarla como producto de relaciones históricas, discursivas y políticas que atraviesan la enfermería contemporánea. Desde esta perspectiva, adoptamos como eje interpretativo el marco foucaultiano, que permite comprender la formación del liderazgo de Grinspun como un proceso de subjetivación y su actuación política e institucional como expresión de técnicas de poder y de gubernamentalidad. Adoptamos aquí la lectura foucaultiana como marco analítico que integra evidencia empírica —segmentos discursivos de la entrevista— y categorías conceptuales (tecnologías disciplinarias, tecnologías del yo, regímenes de verdad, poder-saber, gubernamentalidad), con el fin de explicitar cómo las prácticas y los discursos moldean a los sujetos y las prácticas profesionales. El análisis se organiza en cuatro bloques analíticos: (1) impacto del contexto familiar y de la socialización profesional; (2) transformación de la formación en enfermería en América Latina; (3) innovaciones en investigación, plan de estudios y simulación; y (4) liderazgo institucional y defensa de la profesión: gubernamentalidad, resistencia y expansión de la profesión.

### Impacto del Contexto Familiar, Socialización y Producción de Sujetos en Enfermería

La trayectoria inicial de Grinspun indica que los valores familiares, la movilidad transnacional, las experiencias de superación y el incentivo a la educación constituyeron dispositivos elementales de subjetivación. Según Foucault^(1)^, el sujeto no es un origen autónomo, sino el efecto de prácticas, discursos y relaciones de poder que moldean las formas de ser, actuar y pensar^(2)^. Así, las experiencias relatadas por Grinspun —liderazgo precoz, conciencia social, exposición a contextos de conflicto y educación crítica— funcionan como matrices discursivas que formaron a una profesional orientada hacia la ética, la responsabilidad colectiva y el compromiso político. Más precisamente, estas prácticas familiares y formativas operan como “tecnologías del yo” (technologies of the self): procedimientos mediante los cuales el individuo se constituye, a través de ejercicios de juicio, disciplina y autocuidado, interiorizando normas y desarrollando capacidades de acción política y ética.

La literatura sobre socialización profesional reafirma que las experiencias en la infancia, los desplazamientos geográficos y las situaciones de vulnerabilidad influyen en la constitución de identidades profesionales comprometidas con la justicia social^(13,14)^. Los autores señalan que las trayectorias biográficas a menudo articulan prácticas educativas familiares y formación moral —dimensión explícitamente presente en el relato de Grinspun^(15)^. Desde el punto de vista foucaultiano, la infancia y la juventud no son aquí meros antecedentes temporales, sino dispositivos históricos que inscriben modos de subjetivación: producen predisposiciones para la crítica, para el ejercicio responsable de la autonomía y para la acción colectiva. Así, la constitución de Grinspun como sujeto político viene precedida por una red de prácticas que articulan libertad, disciplina y compromiso ético, y que posteriormente se movilizarán en el ámbito institucional y discursivo de la enfermería.

### Transformación de la Formación en Enfermería en Brasil y en América Latina

El análisis foucaultiano ilumina los procesos macroestructurales que atraviesan la formación profesional. En América Latina, el ámbito formativo se ve afectado por desigualdades, fragmentación curricular y heterogeneidad de estándares^(16)^. La labor de Grinspun se inserta en este contexto como una fuerza que busca desplazar las racionalidades hegemónicas de la enseñanza, acercando la formación y la práctica clínica con base en evidencia internacional^(17)^. Estos desplazamientos configuran operaciones de gubernamentalidad educativa: enuncian normas, métricas y procedimientos que orientan las conductas de estudiantes, docentes y gestores, al mismo tiempo que producen sujetos gobernables (profesionales formados según criterios de eficiencia y seguridad).

La defensa de una educación de calidad, integrada y centrada en competencias dialoga con el debate nacional sobre la articulación entre enseñanza y servicio y la necesidad de planes de estudio críticos y reflexivos^(18)^. En el horizonte foucaultiano, la difusión de estas racionalidades implica la constitución de regímenes de verdad educativos: conjuntos de enunciados y prácticas que definen lo que se considera ”*conocimiento válido*” y ”*buena práctica*” en la formación. Así, la influencia de Grinspun trasciende la mera mejora técnica de los contenidos: reconfigura el régimen discursivo que determina qué se debe enseñar, cómo se debe evaluar y qué tipo de profesional se pretende formar.

### Innovaciones en Investigación, Plan de Estudios y Simulación: Prácticas de Conocimiento y Producción de Verdad

Las innovaciones promovidas por Grinspun —desarrollo curricular, investigación aplicada y uso de simulación realista— articulan prácticas de conocimiento y regímenes de verdad que estructuran la enfermería contemporánea. Para Foucault, los discursos científicos son mecanismos centrales en la producción de la verdad, capaces de legitimar prácticas y orientar conductas. En este sentido, la simulación y la investigación centrada en la práctica no solo transmiten competencias técnicas; constituyen espacios donde se experimentan, validan y difunden enunciados de verdad sobre el cuidado, produciendo sujetos que incorporan dichos saberes en sus prácticas cotidianas.

La creación e implementación de las Guías de Prácticas Óptimas-GPO (en inglés Best Practices Guidelines-BPG) representan un ejemplo paradigmático de dispositivo de poder-saber: las GPO articulan ciencia, política y práctica al traducir la evidencia en estándares y recomendaciones que estandarizan las conductas, reducen la variabilidad en la atención y orientan las decisiones clínicas. Desde la perspectiva foucaultiana, se trata de tecnologías que disciplinan las prácticas al mismo tiempo que pueden promover la autonomía profesional, ya que proporcionan un marco técnico que permite un juicio clínico basado en la evidencia. De este modo, las GPO desempeñan un doble papel: disciplinan mediante normativas e instrumentos de control, y emancipan al ofrecer recursos para la toma de decisiones basada en criterios técnicos y éticos. Este carácter ambivalente merece una atención analítica: ni meramente opresivas ni puramente liberadoras, las prácticas-guía inscriben posibilidades de agencia dentro de los límites institucionales.

### Liderazgo Institucional y Defensa de la Profesión: Gubernamentalidad, Resistencia y Expansión de la Profesión

En la dimensión institucional, Grinspun demuestra un liderazgo que busca transformar estructuras y prácticas. Su labor en la *Registered Nurses’ Association of Ontario* (RNAO) ejemplifica la capacidad de articular políticas, movilizar colectivos e influir en las agendas públicas —prácticas que pueden enmarcarse como formas de gubernamentalidad profesional. Según Foucault, la gubernamentalidad se refiere a las técnicas y racionalidades con las que se orienta el comportamiento de las poblaciones y las instituciones; en este sentido, la construcción de programas, redes y estrategias de defensa por parte de la RNAO constituye un proceso de orientación de las conductas profesionales y de la política de salud.

La literatura indica que la profesionalización exige líderes capaces de disputar los significados de la práctica y promover la justicia social^(19,20)^. Grinspun actúa en este campo fortaleciendo redes y ampliando la visibilidad de la enfermería en los ámbitos políticos, como se evidenció durante la pandemia y en las campañas por la justicia en la distribución de vacunas. Su acción ilustra también el concepto foucaultiano de resistencia: no como mera negación del poder, sino como producción de alternativas —de formas distintas de organización, conocimiento e intervención política que reconfiguran el espacio de las posibilidades institucionales.

La afirmación de Grinspun de que las experiencias de la guerra la convirtieron en ”*activista por la paz*” puede interpretarse como una práctica de constitución ética del sujeto: un ejercicio de tecnología del yo que orienta las decisiones públicas y las formas de compromiso colectivo. Así, su trayectoria institucional opera simultáneamente como producción de conocimiento, ejercicio de poder y producción de subjetividad: un nudo que ejemplifica cómo el liderazgo, la intervención política y la producción de conocimiento se entrelazan para ampliar la gubernamentalidad de la enfermería.

Desde esta perspectiva, el análisis foucaultiano de la trayectoria de Doris Grinspun revela que su potencia como líder proviene de la articulación entre procesos de subjetivación (tecnologías del yo), dispositivos disciplinarios (protocolos, planes de estudio, GPO) y estrategias de gubernamentalidad (acciones institucionales y defensa de causas). Esta articulación muestra que el poder, en la enfermería contemporánea, no se ejerce únicamente como coacción, sino como producción: de conocimientos, de modos de conducta y de sujetos políticos capaces de intervenir en las estructuras que los produjeron.

Por lo tanto, la biografía de Grinspun constituye un material heurístico para comprender cómo los agentes profesionales pueden instaurar desplazamientos discursivos e institucionales que amplían los márgenes de acción, redefinen los regímenes de verdad y promueven cambios en la formación y en las políticas de salud. Desde un punto de vista práctico, esta lectura sugiere que las políticas de formación y gestión deben reconocer la doble dinámica de los dispositivos (disciplinario/emancipatorio) e invertir en estrategias que fomenten tanto la producción de evidencia aplicable como el desarrollo de tecnologías del yo que sustenten la práctica ética y crítica.

Las limitaciones de este estudio se derivan de su diseño histórico-biográfico centrado en la narrativa de una sola líder, obtenida mediante una entrevista virtual e interpretada exclusivamente a la luz del marco foucaultiano, lo que circunscribe el alcance analítico, restringe la triangulación de fuentes y limita la generalización de los hallazgos a otros contextos sociopolíticos de la enfermería global.

## CONCLUSIÓN

El análisis foucaultiano de la trayectoria de Doris Grinspun revela que su liderazgo trasciende los logros individuales y se configura como un dispositivo de transformación capaz de reorganizar las formas de ser, actuar y pensar la enfermería desde una perspectiva global. Al articular la práctica clínica, la gestión de sistemas, la investigación aplicada, la innovación educativa y el activismo político, Grinspun opera en la intersección entre el poder y el saber, contribuyendo a reconfigurar las formaciones, las prácticas y las políticas de salud.

Su legado muestra que la enfermería amplía su gubernamentalidad cuando ocupa posiciones estratégicas en la formulación de políticas, en la producción de evidencia y en la defensa de agendas orientadas al bien común. Esta actuación ilumina caminos especialmente relevantes para los países del Sur Global, poniendo de manifiesto que la ampliación de la autonomía profesional depende de lideranzas capaces de intervenir críticamente en las racionalidades hegemónicas que moldean los sistemas de salud.

Al destacar cómo los dispositivos disciplinarios, las tecnologías del yo y las estrategias de gobernabilidad se articulan en la constitución de su liderazgo, este estudio reafirma la biografía como una herramienta metodológica poderosa para comprender los procesos de cambio profesional. La trayectoria de Doris Grinspun, marcada por el compromiso ético, la visión estratégica y el compromiso político, se configura como una referencia internacional para la construcción de liderazgos críticos, innovadores y transformadores. Por último, se pone de manifiesto que el liderazgo en enfermería es un fenómeno ético-político capaz de producir nuevos regímenes de verdad, transgredir fronteras institucionales y proyectar horizontes ampliados para la profesión a escala global.

Por último, el estudio presenta como limitación la ausencia de triangulación de los resultados con otras fuentes. Sin embargo, se observa la necesidad de explicar, con mayor profundidad analítica, la historia de vida de la biografiada a partir de la entrevista. Este enfoque se presenta como una oportunidad para aplicar el marco filosófico de Michel Foucault, explorando las técnicas del yo en la construcción del sujeto.

## DISPONIBILIDAD DE DATOS

Todo el conjunto de datos que respalda los resultados de este estudio se publicó en el mismo artículo.
